# Copeptin as an Indicator of Hemodynamic Derangement and Prognosis in Liver Cirrhosis

**DOI:** 10.1371/journal.pone.0138264

**Published:** 2015-09-17

**Authors:** Annarein J. C. Kerbert, Len Verbeke, Fang W. T. Chiang, Wim Laleman, Johan J. van der Reijden, Wim van Duijn, Frederik Nevens, Ron Wolterbeek, Bart van Hoek, Hein W. Verspaget, Minneke J. Coenraad

**Affiliations:** 1 Department of Gastroenterology and Hepatology, Leiden University Medical Center, Leiden, the Netherlands; 2 Department of Liver and Biliopancreatic Diseases, University Hospital Gasthuisberg, Leuven, Belgium; 3 Department of Medical Statistics and Bio-Informatics, Leiden University Medical Center, Leiden, the Netherlands; University of Colorado, UNITED STATES

## Abstract

**Background:**

Advanced liver cirrhosis is associated with systemic hemodynamic derangement leading to the development of severe complications associated with increased mortality. Copeptin is a stable cleavage product of the precursor of arginine vasopressin, a key-regulator in hemodynamic homeostasis. Copeptin is currently considered a reliable prognostic marker in a wide variety of diseases other than cirrhosis. The present study aimed to assess copeptin, both experimentally and clinically, as a potential biomarker of hemodynamic derangement and to evaluate its prognostic significance in cirrhosis.

**Materials and Methods:**

Two studies were executed: 1) in 18 thioacetamide-induced cirrhotic rats and 5 control rats, plasma copeptin and hemodynamic measurements were performed, 2) in 61 cirrhotic patients, serum copeptin concentration was measured in samples collected at time of registration at the waiting list for liver transplantation. In 46 patients, also a second copeptin measurement was performed during follow-up while registered at the waiting list for liver transplantation. To determine the association of serum copeptin and clinical data with outcome, Cox proportional hazard regression analysis and Kaplan Meier analysis were performed.

**Results:**

Plasma copeptin concentration was significantly higher in cirrhotic rats than in controls (1.6 ± 0.5 vs. 0.9 ± 0.1 pmol/L, p< 0.01) and was negatively correlated to the mean arterial blood pressure (r = -0.574, p = 0.013). In cirrhotic patients, serum copeptin concentration was high [11.0 (5.2–24.0) pmol/L] and increased significantly during the time of registration at the waiting list for liver transplantation. MELD and MELD-sodium score were significantly correlated to serum copeptin [MELD: (r = 0.33, p = 0.01), MELD-sodium: (r = 0.29, p = 0.02)], also at time of the second copeptin measurement [MELD and MELD-sodium: r = 0.39, p< 0.01]. In cirrhotic humans, serum copeptin concentration was significantly associated with outcome, independently of the MELD and MELD-sodium score. Patients with a low serum copeptin concentration at time of registration at the liver transplant waiting list had significantly better transplant-free survival rates at 3, 6 and 12 months of follow-up as compared to those with a high serum copeptin concentration (Log-rank: p< 0.01, p< 0.01 and p = 0.02 respectively).

**Conclusions:**

Circulating copeptin levels are elevated in rats and humans with cirrhosis. Copeptin is independently associated with outcome in cirrhotic patients awaiting liver transplantation.

## Introduction

Portal hypertension may develop in patients with liver cirrhosis, as a result of an increased intrahepatic vascular resistance, reduced systemic vascular resistance and increased portal inflow. In early stages of cirrhosis, decreases in systemic vascular resistance are compensated by an increase in cardiac output [1, 2]. In more advanced stages, there is a marked reduction of systemic vascular resistance which cannot be compensated by additional increases in cardiac output, leading to a decreased effective arterial blood volume [1]. This triggers the activation of counter regulatory systems, such as the renin-angiotensin-aldosterone (RAAS) system, sympathetic nervous system and non-osmotic release of arginine vasopressin (AVP). Activation of these vasoconstrictor systems helps to restore the effective arterial blood volume, but has negative effects on kidney function, particularly due to renal sodium and solute-free water retention, which is associated with the development of ascites, edema and hyponatremia. Ultimately, intrarenal vasoconstriction and hypoperfusion may lead to the development of a hepatorenal syndrome, which is associated with a poor prognosis [3, 4].

To date, the Model of End stage Liver Disease (MELD) score is widely used as a prognostic score and a tool for organ allocation in patients eligible for liver transplantation (LT) [5]. However, this liver specific score falls short on assessing the severity of circulatory dysfunction. The accuracy of the estimation of prognosis based on information included in liver specific scoring systems, such as the MELD and MELD-sodium (MELD-Na) score, may be improved by adding information on circulatory dysfunction. Because of its key role in circulatory homeostasis and its systemic vasoconstrictor effects [6], AVP might be particularly interesting as a marker of circulatory dysfunction and prognosis in cirrhosis. However, AVP has a relatively short half-life time of approximately 20 minutes and more than 90% of AVP is bound to platelets in the circulation [7]. Therefore, AVP is not useful as a biomarker in clinical practice. Copeptin has been first described in 1972 and is a cleavage product of the C-terminal part of the AVP precursor, pre-pro-vasopressin [8–10], which is secreted by the posterior pituitary in response to hypotension and hyperosmolality [11]. The actual function of copeptin is unknown. In contrast to AVP, copeptin is a stable molecule that does not bind to platelets in the circulation. Moreover, copeptin is secreted together with AVP in equimolar amounts and has a strong correlation with AVP over a wide range of osmolalities [12, 13]. These properties make copeptin an interesting surrogate marker of AVP in clinical practice. Copeptin has been shown to be a reliable prognostic marker in decompensated congestive heart failure [14] and a wide variety of other diseases [15]. To date, limited data are available on its prognostic significance in patients with cirrhosis [16].

In the present study, we hypothesized that serum copeptin concentration would be elevated in the setting of liver cirrhosis accompanied by circulatory dysfunction. In order to test this hypothesis, we performed an animal study with cirrhotic rats that underwent both hemodynamic measurements and serum copeptin measurements. This animal model provided the opportunity to test the ability of copeptin as a surrogate marker of circulatory dysfunction in cirrhosis without interference of therapeutic interventions or the presence of kidney failure. Furthermore, we hypothesized that copeptin would be a predictor of transplant-free survival in cirrhotic humans, in combination with and independently of the MELD and MELD-sodium score. The prognostic potential of copeptin was evaluated, as a proof of principle, in a cohort of cirrhotic patients registered at the waiting list for LT. The specific aims of our study were to evaluate the relationship between circulatory dysfunction and copeptin levels in cirrhotic rats and to evaluate copeptin as an independent predictor of transplant-free survival in cirrhotic humans.

## Materials and Methods

Animal model: Approval for this study was obtained from the ethics committee on animal research of the University Hospital Gasthuisberg, Leuven, Belgium.

Human study: Approval was obtained from the review board of the Leiden University Medical Center, Leiden, the Netherlands. Patient records were anonymized and de-identified prior to analysis. At time of screening for LT, informed consent was obtained to sample and store blood for future research from each patient included in the study.

### Animal model

For this study, 23 age-matched male Wistar rats, weighing 200 to 250 grams, were divided into two groups. Five animals served as a control group and in 18 animals liver cirrhosis was induced by adding thioacetamide (TAA, Sigma-Aldrich N.V., Bornem, Belgium) to their drinking water according to protocol [17]. Initially, a concentration of 0.03% of TAA in drinking water was used. Subsequently, concentrations were adapted weekly depending on individual body weight. Practically, TAA concentrations were increased (decreased) with 50% if the weight increased (decreased) more than 20 g weekly or if the overall weight increased (decreased) more than 60 g [17]. At 18 weeks, the carotid artery and portal vein were cannulated for measurement of portal venous pressure, mean arterial blood pressure (MAP) and mesenteric blood flow (MBF) [17]. MBF was determined via a 1 millimeter non-constrictive perivascular flow probe (1RB, Transonic, Ithaca, NY) connected to a T-206 flowmeter. Blood samples were collected in heparinized tubes (BD Vacutainer®) by puncturing the aortic bifurcation for the measurement of plasma copeptin levels and routine biochemical analysis. To confirm cirrhosis in the animals, liver tissue was collected and fixed in formaldehyde 6% solution, embedded in paraffin and stained with Sirius-red.

### Human study

The population of this retrospective study consisted of 61 patients with cirrhosis, who were screened and registered at the waiting list for LT at the Leiden University Medical Center (LUMC), a tertiary referral center. For all patients, a blood sample for serum copeptin measurement was available in the institutional’s biobank, which was obtained at time of registration at the waiting list and stored at -20°C. Median (IQR) time interval between drawing of the blood sample and time of registration at the waiting list was 1.0 (-7.5–3.0) day. For 46 patients, a second serum sample for copeptin measurement was available, obtained during the time of registration at the waiting list for LT. Median (IQR) time interval between the two measurements was 156 (112–283) days.

Patient demographics, clinical characteristics and laboratory measurements (INR and serum sodium, bilirubin, albumin and creatinine concentration) were retrieved from patient files. The MELD, MELD-Na and Child-Pugh score were calculated based on these laboratory results and clinical findings [5, 18].

### Laboratory analysis

Measurement of plasma copeptin concentration in animal samples was performed using ELISA assay for rat copeptin according to manufacturer’s instructions (NovaTeinBio, Inc, Cambridge, MA).

Copeptin concentration in human serum was determined using an assay in chemiluminescence-coated tube format (B.R.A.H.M.S., Kryptor, GmbH, Henningsdorf, Germany). Median plasma copeptin concentration in 359 healthy individuals was 4.2 (1.0–13.8) pmol/L [19]. The intra-assay variability was < 9% and < 8% for copeptin concentrations > 20 pmol/L and > 50 pmol/L, respectively. The inter-assay variability found in our measurements was < 19% for copeptin concentrations > 20 pmol/L and < 14% for concentrations > 50 pmol/L. The investigators were blinded for clinical outcomes.

### Statistical analysis

Differences in copeptin concentration between groups were tested for significance using the Kruskal Wallis test, Wilcoxon rank sum test or Student’s t-test when appropriate. A Pearson bivariate correlation analysis was performed to correlate copeptin concentrations to MAP, MBF, portal venous pressure and body weight in rats. Pearson bivariate correlation analysis and Spearman’s rank order correlation analysis were performed when appropriate to determine possible correlations between copeptin, routine biochemical markers and MELD and MELD-Na scores in humans.

Transplant-free survival analysis at 3, 6 and 12 months of follow-up, stratified according to serum copeptin concentration, MELD and MELD-Na score at admission was performed using Kaplan Meier analysis and compared using the Log-rank test. Cox proportional hazard regression analysis was performed using ‘LT or death awaiting LT’ as a combined endpoint at 3, 6 and 12 months of follow-up. Optimal cut-off points of serum copeptin, MELD score and MELD-Na score in predicting LT or death awaiting LT at 3, 6 and 12 months of follow-up were determined using the Youden index. Hereinafter, values exceeding these optimum cut-off points are referred to as ‘high’ and values below these cut-off points as ‘low’. These factors and the MAP were included in univariate analysis. Variables with a p< 0.20 in univariate analysis were included in multivariate analysis. Discrete variables are shown as counts (percentage) and continuous variables as mean (± standard deviation). Data with a skewed distribution are expressed as median [interquartile range (IQR)]. P≤ 0.05 was considered statistically significant.

## Results

### Animal model

No mortality was seen at 18 weeks of TAA intoxication or in control rats. Baseline clinical and hemodynamic characteristics of the intervention and control group are shown in Table 1. All animals in the intervention group had developed macroscopic and microscopic cirrhosis. No animal had renal failure, one animal had ascites. In the cirrhotic rats, significantly higher plasma copeptin concentrations were found than in the control rats (1.6 ± 0.5 vs. 0.9 ± 0.1 pmol/L, p = 0.01). Cirrhotic rats had a significantly lower body weight and MAP as compared to control rats. As expected, cirrhotic rats gained less weight as compared to the control rats during the study due to the induced liver injury. Portal venous pressure and MBF per 100 grams body weight were significantly higher in cirrhotic rats than in controls. Plasma copeptin concentration was negatively correlated to MAP (r = -0.57, p = 0.01) and to body weight (r = -0.57, p = 0.02). No significant correlation was found between copeptin and portal venous pressure (r = 0.32, p = 0.10) or MBF (r = 0.03, p = 0.91).

**Table 1:** Clinical and hemodynamic characteristics of cirrhotic rats and control rats at 18 weeks.

Variable	Cirrhotic rats (n = 18)	Control rats (n = 5)	p-value
Copeptin (pmol/L)	1.6 ± 0.5	0.9 ± 0.1	0.01
MAP (mmHg)	70 ± 17	137 ± 4	< 0.01
MBF (ml/min/100g)	5.1 ± 1.1	2.5 ± 1.2	< 0.05
Portal pressure (mmHg)	10.5 ± 2.2	5.6 ± 0.5	< 0.01
Body weight (g)	337 ± 49	524 ± 79	< 0.01

*MAP*, *mean arterial blood pressure; MBF*, *mesenteric blood flow*.

### Human study

Demographics, clinical characteristics and biochemical parameters at time of registration at the LT waiting list of 61 patients with liver cirrhosis are shown in Table 2. Median serum copeptin concentration at baseline was 11.0 (5.2–24.0) pmol/L. No effect of gender on serum copeptin concentration was found [males: 12.3 (5.8–28.5) pmol/L vs. females: 10.5 (4.4–21.0) pmol/L, p = 0.42]. In the 46 patients with a second serum sample available while on the waiting list, median serum copeptin concentration in the second sample was significantly higher as compared to the baseline serum copeptin concentration [20.3 (10.0–37.6) pmol/L vs. 10.8 (4.8–23.8) pmol/L, p< 0.01]. MELD and MELD-Na score did not significantly change in the time period between the first and second copeptin measurement [MELD: 13.8 (11.4–16.9) vs. 14.0 (12.0–17.5), p = 0.66 and MELD-Na: 14.9 (12.0–17.6) vs. 15.0 (13.0–20.0), p = 0.14]. Serum creatinine concentration was also stable over time [88.5 (67.8–110.5) vs. 88.5 (71.0–123.0) μmol/L, p = 0.28].

**Table 2:** Demographic and clinical characteristics of 61 cirrhotic patients at time of registration at the waiting list for liver transplantation.

Variable	All patients (n = 61)
**Age, years**	54 (43–60)
**Male, n (%)**	46 (75.4)
**Etiology of cirrhosis, n (%)**	
HBV or HCV	11 (18.0)
Alcohol	21 (34.4)
Hepatitis + alcohol	6 (9.8)
PSC or PBC	11 (18.0)
AIH	4 (6.6)
Cryptogenic	4 (6.6)
NASH	3 (4.9)
Budd Chiari	1 (1.6)
**HCC, n (%)**	5 (8.2)
**Ascites, n (%)**	41 (67.2)
**Diuretic use** ^§^ **, n (%)**	41 (67.2)
Spironolactone alone	10 (16.4)
Furosemide alone	2 (3.3)
Spironolactone + furosemide	29 (47.5)
**Betablocker use, n (%)**	25 (41.0)
**Child Pugh class, n (%)**	
A	10 (16.4)
B	32 (52.5)
C	19 (31.1)
**MELD score**	13.5 (11.4–17.0)
**MELD-Na score**	15.0 (12.0–18.2)
**Copeptin (pmol/L)**	11.0 (5.2–24.0)
**Sodium (mmol/L)**	138 (136–141)
**Creatinine (μmol/L)**	86 (69–109)
**Bilirubin (μmol/L)**	45 (27–85)
**Albumin (g/L)**	32 (28–36)
**INR**	1.3 (1.2–1.4)
**MAP** ^†^ **(mmHg)**	84.7 (80.0–93.3)

*HBV*, *hepatitis B virus; HCV*, *hepatitis C virus; PSC*, *primary sclerosing cholangitis; PBC*, *primary biliary cirrhosis; AIH*, *autoimmune hepatitis; NASH*, *non-alcoholic steatohepatitis; HCC*, *hepatocellular carcinoma; MELD*, *Model for End-Stage Liver Disease; MELD-Na*, *sodium MELD; INR*, *International Normalized Ratio; MAP*, *mean arterial blood pressure*

Data are shown as counts (percentage) or as median (interquartile range).

^§^ Median (interquartile range) doses of diuretics used at baseline: spironolactone 100.0 (50.0–187.5) mg/day, furosemide 40.0 (40.0–70.0) mg/day.

^†^ MAP was determined in 55 patients.

Significant positive correlations were found between baseline serum copeptin concentration and serum creatinine concentration (r = 0.30, p = 0.02), MELD score (r = 0.33, p = 0.01), MELD-Na score (r = 0.29, p = 0.02) and INR (r = 0.27, p = 0.04). No correlations were found between baseline serum copeptin concentration and serum bilirubin concentration (r = 0.12, p = 0.36), serum sodium concentration (r = -0.02, p = 0.89) and MAP (r = 0.01, p = 0.94). There were no significant differences in serum copeptin concentration at time of registration at the waiting list between patients with or without ascites [13.5 (6.3–28.9) vs. 7.9 (3.7–18.8), p = 0.13] or with or without the use of diuretics [10.6 (5.2–26.2) vs. 11.0 (4.8–23.4), p = 1.00]. In the 46 patients in whom a second copeptin measurement was performed while at the waiting list for LT, significant positive correlations were found between the second serum copeptin concentration and the MELD score (r = 0.39, p< 0.01) and MELD-Na score (r = 0.39, p< 0.01) at time of sampling of the second blood sample.

**Fig 1:**
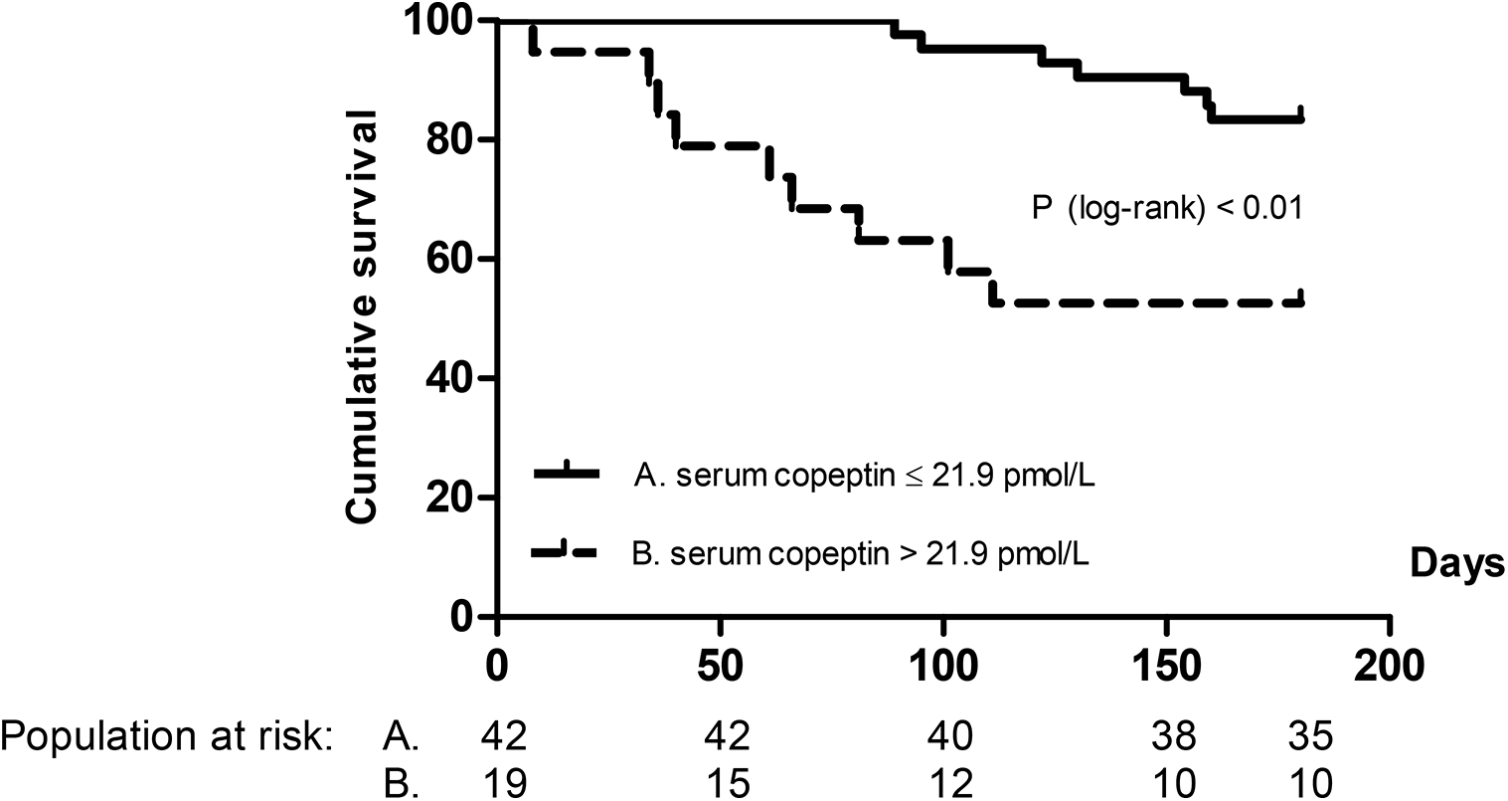
Association of serum copeptin concentration with transplant-free survival. Kaplan Meier survival analysis at 6 months of follow-up of 61 cirrhotic patients registered at the waiting list for liver transplantation, stratified according to serum copeptin concentration (pmol/L) at time of registration.

Median (IQR) time interval between time of registration at the waiting list for LT and death (n = 10) or LT (n = 50) was 274 (183–453) days. One patient survived without a LT. After one year of follow-up, 5 patients had died and 35 were transplanted (3 months: 1 patient died, 7 patients transplanted; 6 months: 2 patients died, 14 patients transplanted). Transplant-free survival at 6 months of follow-up, stratified according to serum copeptin concentration at time of registration at the waiting list, is shown in Fig 1. Patients with a low serum copeptin concentration had a significantly better transplant-free survival than patients with a high serum copeptin concentration (Log-rank: p< 0.01). Also at 3 and 12 months of follow-up, patients with a low serum copeptin concentration had a significantly better outcome as compared to those with a high serum copeptin concentration (Log-rank: p< 0.01 and p = 0.02, respectively).

Results of univariate and multivariate Cox proportional hazard regression analyses assessing the association of high serum copeptin concentrations and high MELD and MELD-Na scores with outcome at 6 and 12 months of follow-up are shown in Table 3. Results at 3 months of follow-up were comparable to those at 6 months of follow-up (data not shown), despite of a relatively low number of events at this time point (n = 8).

**Table 3:** Parameters associated with survival time. Optimal cut-off points and results of univariate (A) and multivariate (B) Cox proportional hazard regression analysis assessing factors associated with liver transplantation or death awaiting liver transplantation in relation to time of follow-up in cirrhotic patients registered at the waiting list for liver transplantation (n = 61).

Variable	6 months		12 months	
Death or LT, n (%)	16 (26.2)		40 (65.6)	
Copeptin cut-off (pmol/L)	21.9		21.9	
MELD score cut-off (points)	17		13	
MELD-Na score cut-off (points)	17		20	
**A. Univariate**	**HR (95% CI)**	**p-value**	**HR (95% CI)**	**p-value**
High serum copeptin	4.1 (1.5–11.0)	< 0.01	2.1 (1.1–4.0)	0.02
High MELD score	3.5 (1.3–9.5)	0.01	2.3 (1.2–4.3)	0.01
High MELD-Na score	3.5 (1.3–9.3)	0.01	3.7 (1.9–7.5)	< 0.01
**Copeptin and MELD-score** ^§^				
Low copeptin and high MELD	3.5 (0.8–15.8)	0.10	2.2 (1.0–5.1)	0.06
High copeptin and low MELD	4.1 (1.1–15.3)	0.04	2.4 (0.8–7.5)	0.14
High copeptin and high MELD	13.5 (3.3–54.6)	< 0.01	3.3 (1.5–7.4)	< 0.01
**Copeptin and MELD-Na score** ^†^				
Low copeptin and high MELD-Na	2.2 (0.5–10.0)	0.29	2.8 (0.8–9.6)	0.09
High copeptin and low MELD-Na	4.1 (1.0–17.4)	0.05	1.5 (0.7–3.4)	0.33
High copeptin and high MELD-Na	13.7 (3.0–61.7)	< 0.01	4.9 (2.2–11.1)	< 0.01
**B. Multivariate**	**HR (95% CI)**	**p-value**	**HR (95% CI)**	**p-value**
**Model 1**				
High serum copeptin	4.0 (1.5–10.7)	< 0.01	1.7 (0.9–3.3)	0.12
High MELD	3.4 (1.3–9.2)	0.02	1.9 (1.0–3.8)	0.07
**Model 2**				
High serum copeptin	3.4 (1.2–9.3)	0.02	1.6 (0.8–3.1)	0.20
High MELD-Na	2.7 (1.0–7.5)	0.05	3.1 (1.5–6.6)	< 0.01

*LT*, *liver transplantation; HR*, *hazard ratio; 95% CI*, *95% confidence interval; high*, *exceeding the optimal cut-off point; low*, *equal to or below the optimal cut-off point; MELD*, *Model for End-Stage Liver Disease; MELD-Na*, *sodium MELD*

^§,†^ “Low” and “high” refers to values below and above the optimal cut-off point as defined using the Youden index, respectively. The reference groups were patients with low serum copeptin and ^§^low MELD score or ^†^low MELD-Na score.

In the univariate analyses, a high serum copeptin concentration, high MELD score and high MELD-Na score at time of registration at the waiting list were significantly associated with the combined endpoint ‘LT or death awaiting LT’ at 6 and 12 months of follow-up. No significant association with the combined endpoint at these time points was found for MAP at enlistment [3 months: HR = 1.00 (95% CI = 0.94–1.06), p = 0.86; 6 months: HR = 1.02 (95% CI = 0.98–1.95), p = 0.48; 12 months: HR = 1.02 (95% CI = 0.98–1.05), p = 0.44].

In the multivariate analyses, a high serum copeptin concentration at enlistment was significantly associated with the combined endpoint, independently of a high MELD- or MELD-Na score at 6 months of follow-up. At 12 months of follow-up, there was no significant association of a high serum copeptin concentration at time of registration at the waiting list with the combined endpoint, when adjusted for high MELD- or MELD-Na scores.

**Fig 2:**
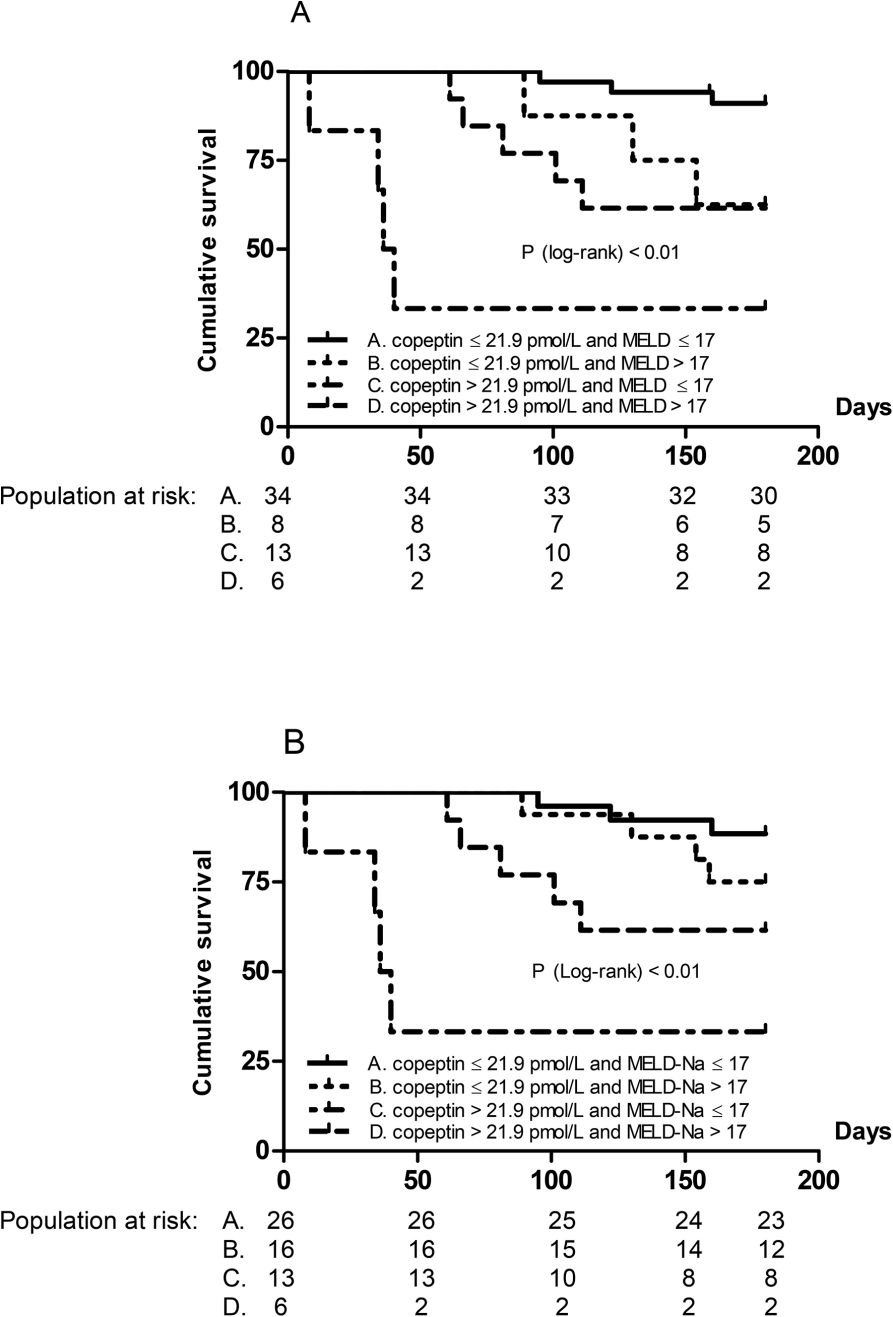
Association of serum copeptin concentration and MELD and MELD-Na score with transplant-free survival. Kaplan Meier survival analysis at 6 months of follow-up of 61 cirrhotic patients registered at the waiting list for liver transplantation, stratified according to serum copeptin concentration (pmol/L) and MELD score (A) and serum copeptin concentration and MELD-Na score (B) at time of registration.

The subgroup of patients with both a low serum copeptin concentration and low MELD or MELD-Na score at time of registration at the waiting list displayed the best transplant-free survival rates at 6 and 12 months of follow-up as compared to patients with both a high serum copeptin concentration and high MELD or MELD-Na score. The other two subgroups composed based on either a high or low serum copeptin concentration and MELD or MELD-Na score at baseline, showed an intermediate transplant-free survival rate (Table 3A). Fig 2A and 2B show transplant-free survival curves at 6 months of follow-up stratified for both serum copeptin concentration and MELD score and serum copeptin concentration and MELD-Na score at time of registration at the waiting list, respectively. Combining the two subgroups with either a high or low serum copeptin concentration or MELD or MELD-Na score resulted in similar univariate Hazard ratios at 6 and 12 months for these groups, i.e. 3.9 (95% CI = 1.2–12.8) and 2.3 (95% CI = 1.0–4.9) for the copeptin/MELD score combination (both p< 0.05), and 3.0 (95% CI = 0.8–11.1) and 1.7 (95% CI = 0.8–3.6) for the copeptin/MELD-Na score combination (both p< 0.15), respectively.

## Discussion

The results of the present study show that copeptin, as a surrogate marker of circulatory dysfunction, is elevated in both cirrhotic rats and humans, and that copeptin is an independent predictor of 6-month transplant-free survival in cirrhotic humans.

We investigated the relationship between copeptin and hemodynamic characteristics in an animal model bearing strong resemblance to human cirrhosis, including typical features of portal hypertension and the hyperdynamic circulation, but with a normal kidney function as shown previously [17] and without the use of therapeutic interventions which may affect copeptin levels. In this animal model, plasma copeptin concentration was significantly higher in cirrhotic animals as compared to controls and copeptin was negatively correlated with MAP, thereby confirming our hypothesis. No relationship between copeptin and portal pressure or mesenteric blood flow was found in these cirrhotic animals. It has previously been shown that copeptin is extracted in the kidneys in cirrhotic humans [20] and several previous studies have shown an inverse correlation between copeptin concentration and renal function [21–23]. In the present human study, these findings were confirmed by a significant inverse correlation of serum copeptin with creatinine, but the study design of the present and previous studies was not appropriate to define whether an increase in serum copeptin is causally related to renal impairment. The results of the animal study add to these data that serum copeptin concentration is elevated in cirrhotic rats with portal hypertension and circulatory dysfunction, even in the absence of kidney failure, ascites and the use of medication.

In cirrhotic humans, previous studies have been performed to test the association of copeptin with circulatory dysfunction. It has been shown that copeptin is positively correlated with portal pressure [24] and inversely correlated with cardiac output [20] in cirrhosis. As copeptin has been found to be a potential marker of development of cardiac dysfunction [24], which is associated with poor prognosis in cirrhosis [25], we and others hypothesized that copeptin might also give prognostic information in cirrhosis [16, 24]. The prognostic potential of copeptin as a surrogate marker of circulatory dysfunction has already been demonstrated in the setting of acute myocardial infarction and congestive heart failure [26–28]. To date, two studies evaluated the prognostic significance of copeptin in the setting of liver cirrhosis. Moreno et al [16] showed that copeptin independently predicted 1-year mortality or LT in cirrhotic patients. In contrast, Wiese et al [24] did not find copeptin to be related to long-term survival. To the best of our knowledge, there are no studies specifically assessing the prognostic value of copeptin in a population of cirrhotic patients registered at the waiting list for LT. Currently, the MELD-score is widely used as an organ allocation tool in patients registered at the waiting list for LT and as a prognostic tool in patients undergoing therapy such as transjugular intrahepatic portosystemic shunt (TIPS) procedure [5, 29]. The MELD score characterizes the severity of the underlying liver disease and kidney function, but falls short on assessing the severity of portal hypertension associated with circulatory dysfunction. The MELD-Na score has also been proposed as a marker for organ allocation [18]. In this score, serum sodium is accounting for hemodynamic deregulations associated with end-stage cirrhosis. Incorporation of sodium in the MELD score has been shown to improve its prognostic accuracy [18]. However, a limitation of the MELD-Na score is that marked changes in serum sodium may result from several factors, such as administration of diuretics and hypotonic fluids. Several studies have shown that parameters estimating systemic hemodynamics have a better prognostic ability in predicting survival in cirrhosis than those assessing liver function [30–32]. Therefore, we hypothesized that markers of hemodynamic dysfunction would be predictors of transplant-free survival in cirrhosis, independently of widely used liver specific prognostic scoring systems. However, the assessment of the presence and impact of hemodynamic dysfunction in cirrhosis is complicated, due to the instability and poor reproducibility of potential biomarkers such as plasma norepinephrine, renin activity and AVP concentration. AVP is particularly interesting as a marker of circulatory dysfunction and prognosis in cirrhosis as it is not only a potential biomarker, but is also involved in the pathogenesis of the development of complications of cirrhosis, due to its systemic vasoconstrictor effects. Copeptin, the surrogate marker of AVP, is easily applicable in clinical practice and therefore interesting as a marker of hemodynamic derangement and prognosis in cirrhosis.

In the present study, we found that a high serum copeptin concentration at time of registration at the waiting list for LT was associated with a poor 6-month outcome as defined by LT or death awaiting LT, independently of high MELD- and MELD-Na scores in cirrhotic patients. The independent prognostic value of copeptin disappeared after 12 months of follow-up. This might be explained by changes in disease course over time, and partially by the limited number of patients included in this study. In addition, we showed that copeptin provides additional prognostic information to the MELD and MELD-Na score as patients with both a low MELD or MELD-Na score and low copeptin concentration displayed a significant better outcome as compared to patients with both a high MELD or MELD-Na score and high serum copeptin concentration and patients with either a high or low score and serum copeptin concentration.

A few limitations regarding the animal and human study are to be considered. In the animal study, copeptin concentrations showed a significant, negative correlation with MAP, whereas no significant correlation with MAP was found in the human study. There are several possible explanations for this finding. MAP is influenced by numerous exogenous factors, such as age, body position and methods of measurement. These factors were better controlled in the animal model as compared to the retrospectively enrolled patient population. Moreover, the patient population was more heterogeneous, consisting of patients with diverse stages of the underlying liver disease and presence of clinical decompensation, which required treatment with diuretics or non-selective beta-blockers. In addition, the majority of patients included had Child Pugh A or B cirrhosis and it may be difficult to point out a correlation between MAP and copeptin in more early stages of liver dysfunction. Considering all factors mentioned above, the results obtained in the animal study cannot be directly extrapolated to a population of cirrhotic humans. Another limitation of the present human study is the fact that it is a single centre study with a limited number of patients. One of the consequences of this small study population is a relatively low number of events in survival analysis at 3 months of follow-up. Furthermore, copeptin and other laboratory measurements were not sequentially performed at set time-points due to the retrospective study design. However, in patients who had a follow-up sample available for copeptin measurement while registered at the waiting list for LT, it was found that serum copeptin significantly increased over time. This second copeptin concentration was significantly correlated to the MELD and MELD-Na score at that time-point, whereas no significant increase in MELD and MELD-Na score was found. Also serum creatinine did not increase over time, which suggests that the significant increase in serum copeptin is not related to deterioration of kidney function. However, it needs to be considered that there was a wide variety in time intervals between the index and second copeptin measurement in these 46 patients. Prospective studies in which serum copeptin and other biomarkers of hemodynamic derangement are sequentially performed at set time points are needed to gain more knowledge about the relation between serum copeptin changes over time and the course of hemodynamic derangement, disease progression and survival in patients with stable cirrhosis and in those with acute decompensation of cirrhosis. Furthermore, studies in larger cohorts of cirrhotic patients eligible for LT are needed to validate the results found in the present study and to explore whether copeptin might actually improve the prognostic accuracy of the MELD and MELD-Na score in predicting mortality and the need for a LT.

In conclusion, circulating copeptin concentrations are elevated in both cirrhotic rats and cirrhotic patients. Moreover, the results of the present study show, as a proof of principle, that copeptin is associated with outcome independently of both the MELD and MELD-Na score in a cohort of patients registered at the waiting list for LT and could be useful in combination with liver specific prognostic scores for a more accurate assessment of prognosis.

## Abbreviations:

ADH: antidiuretic hormone
AIH: autoimmune hepatitis
AVP: arginine vasopressin
HCC: hepatocellular carcinoma
HR: hazard ratio
HVPG: hepatic venous pressure gradient
INR: international normalized ratio
IQR: interquartile range
LT: liver transplantation
MAP: mean arterial blood pressure
MBF: mesenteric blood flow
MELD: model for end-stage liver disease
MELD-Na: sodium MELD
NASH: non-alcoholic steatohepatitis
PBC: primary biliary cirrhosis
PSC: primary sclerosing cholangitis
RAAS: renine-angiotensin-aldosterone system
TAA: thioacetamide
TIPS: transjugular intrahepatic portosystemic shunt
95% CI: 95% confidence interval
